# Mechanical Stretching-Induced Traumatic Brain Injury Is Mediated by the Formation of GSK-3β-Tau Complex to Impair Insulin Signaling Transduction

**DOI:** 10.3390/biomedicines9111650

**Published:** 2021-11-09

**Authors:** Pei-Wen Cheng, Yi-Chung Wu, Tzyy-Yue Wong, Gwo-Ching Sun, Ching-Jiunn Tseng

**Affiliations:** 1 Department of Education and Research, Kaohsiung Veterans General Hospital, Kaohsiung 813414, Taiwan; wongtzyyyue@gmail.com (T.-Y.W.); cjtseng@vghks.gov.tw (C.-J.T.); 2 Department of Biomedical Science, National Sun Yat-sen University, Kaohsiung 80424, Taiwan; 3 Section of Neurology, Zuoying Branch of Kaohsiung Armed Forces General Hospital, Kaohsiung 81342, Taiwan; m870563@yahoo.com; 4 School of Medicine, National Defense Medical Center, Neihu, Taipei 11490, Taiwan; 5 International Center for Wound Repair and Regeneration, National Cheng Kung University, Tainan 70101, Taiwan; 6 Department of Anesthesiology, Kaohsiung Medical University Hospital, Kaohsiung 80756, Taiwan; gcsun39@yahoo.com.tw; 7 Faculty of Medicine, College of Medicine, Kaohsiung Medical University, Kaohsiung 80708, Taiwan

**Keywords:** mechanical stimulations, traumatic brain injury (TBI), stretching, neurons, GSK-3β, p-Tau

## Abstract

Traumatic brain injury confers a significant and growing public health burden. It is a major environmental risk factor for dementia. Nonetheless, the mechanism by which primary mechanical injury leads to neurodegeneration and an increased risk of dementia-related diseases is unclear. Thus, we aimed to investigate the effect of stretching on SH-SY5Y neuroblastoma cells that proliferate in vitro. These cells retain the dopamine-β-hydroxylase activity, thus being suitable for neuromechanistic studies. SH-SY5Y cells were cultured on stretchable membranes. The culture conditions contained two groups, namely non-stretched (control) and stretched. They were subjected to cyclic stretching (6 and 24 h) and 25% elongation at 1 Hz. Following stretching at 25% and 1 Hz for 6 h, the mechanical injury changed the mitochondrial membrane potential and triggered oxidative DNA damage at 24 h. Stretching decreased the level of brain-derived neurotrophic factors and increased amyloid-β, thus indicating neuronal stress. Moreover, the mechanical injury downregulated the insulin pathway and upregulated glycogen synthase kinase 3β (GSK-3β)^S9^/p-Tau protein levels, which caused a neuronal injury. Following 6 and 24 h of stretching, GSK-3β^S9^ was directly bound to p-Tau^S396^. In contrast, the neuronal injury was improved using GSK-3β inhibitor TWS119, which downregulated amyloid-β/p-Taus396 phosphorylation by enhancing ERK1/2T202/Y204 and AktS473 phosphorylation. Our findings imply that the neurons were under stress and that the inactivation of the GSK3β could alleviate this defect.

## 1. Introduction

Over 60 million new cases of traumatic brain injury (TBI) occur every year worldwide. TBI is the leading cause of death and disability in people aged 1–40 years [1,2,3]. Despite technological advancements, the treatment for TBI has remained static and is limited to palliative care. The insults leading to TBI include sports injury, crash, falls, and assault [4]. The impact of brain injury can be permanent and can affect an individual in several ways, such as cognition impairment and personality change [4]. The impact is usually negative, which can further affect society. Therefore, it is important to understand the kind of strain that leads to TBI. Moreover, following TBI, even mild physical strain becomes sustained in the neurons. Recent studies have associated TBI with the pathologic accumulation of the neurotoxic proteins Tau, TDP-43, and amyloid-beta, leading to progressive neurodegenerative diseases, including chronic traumatic encephalopathy [5], Alzheimer’s disease (AD) [6] and other dementias [7,8]. A moderate to severe TBI is sufficient to increase the risk of developing dementia up to four-fold [6]. Despite these findings, the mechanism by which primary mechanical injury leads to neurodegeneration and increases the risk of dementia-related diseases is unclear. Identifying the interplay between environmental and genetic risk factors for neurodegenerative diseases is critical for the development of therapeutics to mitigate and prevent subsequent pathology. Recently, researchers have attempted the development of in vitro TBI models using a bio-inspired mechanic device. Previous studies have used a high-frequency device to stretch the neurons [9]. This resulted in an injury to the cell bodies and axons. The neurons were found to grow and survive with the rearrangement of cytoskeleton proteins, particularly microtubules, on being subjected to 5% strain. In addition, the aforementioned strain value enhanced the velocity of the synaptic vesicles. On the contrary, an aggressive strain, such as 50% [10], can change the MMP [11], release lactate dehydrogenase, and cause cell death [12,13]. Considering the aforementioned outcomes, mechanical stimulation can alter cell physiology. Therefore, mechanical stimulation can be used for the development of in vitro TBI models based on their impact on neuron growth and survival. Nonetheless, the field of mechanical stimulation on neurons is still unclear.

Reactive oxygen species (ROS) are excessively generated in the injured brain tissue during and after TBI. This can be attributed to changes in the oxygen demand and an abnormal accumulation of cells susceptible to lipid oxidation [14]. Together with the inflammatory response, oxidative stress is supposedly the culprit that leads to neurodegenerative diseases. The aforementioned AD biomarkers can be used to study their expressions and to achieve an in vitro TBI model. Amyloid-β (Aβ) peptides generated from amyloid precursor protein are the hallmark of AD. Aβ peptides accumulate in the brain tissue of patients with AD [15]. The accumulation of Aβ peptides can affect insulin signaling [16], synaptic function, and aberrant gene expressions. In addition, the aforementioned abnormal accumulation plays a role in memory loss. Previous studies contain illustrations representing the diseased neurons with increased Aβ peptides, hyperphosphorylated Tau, alpha-synuclein, and polyglutamine [17]. Furthermore, Tau (or microtubule-associated protein Tau) and α-synuclein proteins are aggregated in AD and PD brains [18,19,20]. Besides, the change in metabolism following TBI includes an impairment of insulin signaling. Insulin and insulin growth factor (IGF) signaling in the central nervous system regulate cognitive function [21,22]. Insulin/IGF resistance in AD brain leads to decreased phosphoinositol-3-kinase (PI3K)/Akt [23,24] and Wnt/β-catenin signaling [25], as well as increased activation of glycogen synthase kinase 3β (GSK-3β). The impaired insulin/IGF signaling can also disrupt Aβ peptide accumulation [26]. In consequence, the metabolism changes and abnormal protein accumulation promotes oxidative stress in the neurons.

The release of Tau in the cerebrospinal fluid and blood is reportedly a sign of axonal injury. The level of Tau protein increases in Olympic boxers following a mild trauma to the head [27]. Previous studies reported that Tau increase possibly indicates an injury to the central nervous system [28]. The aggregated Tau protein is reportedly toxic, thus was implicated in forming the association between TBI and neurodegenerative diseases. The Tau protein becomes hyperphosphorylated and forms an abnormal aggregate in the cell body of a neuron [21]. It is involved in modulating the binding of microtubule proteins. Thus, the abnormal aggregation of Tau disrupts microtubule formation. This, in turn, disrupts the transmission of neuronal signals and vesicle trafficking in the neurons. We aimed to investigate if mechanical stretching induces oxidative stress and mitochondrial membrane potential (MMP) that causes insulin signaling defects under pathological conditions. Moreover, we intended to identify events critical to the development of Aβ and Tau aggregation. In addition, we clarified if a defect in neuronal insulin signaling triggers the formation of p-Tau^S396^ and pGSK3β^S9^ complex, thus implying neuronal stress. Overall, our results suggest that this neuronal insulin signaling defect is a core mechanism that induces a form of p-Tau^S396^ and pGSK3β^S9^ complex, implying that the neuron cells were under stress and that inactivation of the GSK3β may alleviate this defect.

## 2. Methods

### 2.1. Cell Culture

SH-SY5Y neuroblastoma cells were used in this study. The SH-SY5Y cells are frequently used for studying neuron behavior affected by neurotoxic and mechanical injury. The SH-SY5Y cells were maintained in an undifferentiated state. Previously, differentiated SH-SY5Y cells were found resistant to oxidative stress with altered mitochondrial function. The cells were maintained in DMEM, supplemented with 10% FBS, 100 μg/mL penicillin-streptomycin at 37 °C, and 5% CO_2_. They were seeded on polydimethylsiloxane (PDMS) at a density of 1.5 × 10^4^/cm^2^. The PDMS membrane was stretchable and transparent. 

### 2.2. Stretch Device

The stretch device comprised the following two parts: (1) a primary unit with a strain spindle and (2) a side tray with chambers for cell culture. The cells were cultured in the chamber, filled with a complete medium on the side tray. The side tray was attached to the primary unit with a controlled strain amount. The cells attached to the PDMS membrane were stretched in the biaxial direction. While one end was fixed to the clip, the other end was being stretched.

### 2.3. Stretched-Injury Model

The cells were stretched following their complete attachment to the PDMS surface. The latter was pre-coated with collagen at 24 h following the seeding. Based on a previous study on moderate and severe stretch-injury model, the neurons were subjected to a severe mechanical stretch comprising a rapid onset strain pulse (25% membrane deformation for 6 and 24 h) at a frequency of 1 Hz. The stretching was performed in a humidified atmosphere at 37 °C and 5% CO_2_.

### 2.4. Cell Alignment Measurement

After stretching, the cells were observed under the microscope (Leica Camera Incorporation, Wetzlar, Germany) with a bright field. Images were taken randomly at magnification 200×. Five fields of view were analyzed, with each field of view having at least 30 cells. The cell orientation or alignment was analyzed using ImageJ software (National Institute of Health, Bethesda, MD, USA) with the angle analysis tool. Cells with angles equal to and greater than 30°, or equal to and less than −30° were categorized as aligned cells. The cells that were between 30 and −30° were categorized as not aligned.

### 2.5. Immunoblotting Assay

We resolved the protein (30 μg/sample assessed by the bicinchoninic acid protein assay, Pierce Chemical Co., Rockford, IL, USA) on a 6% polyacrylamide gel and transferred them to a polyvinylidene fluoride membrane (GE Healthcare, Buckinghamshire, UK). The membranes were incubated with appropriate anti-p-TauT231 (ab151559), anti-P-AktS473 (4060, Cell Signaling Technology, Beverly, MA, USA), anti-P-GSK-3βS9 (05-643, EMD Millipore, Billerica, MA, USA), anti-amyloid precursor protein (ab12266), anti-Tau (ab80579), anti-Akt (9272), anti-GSK-3β (07-389), or anti-BDNF antibodies. All the primary antibodies were used at the dilution of 1:1000 in PBST with 5% bovine serum albumin. They were then incubated in an HRP-labelled goat anti-rabbit secondary antibody at 1:10,000. We developed the membranes using an ECL-Plus detection kit (GE Healthcare).

### 2.6. Measuring BDNF Levels

We measured the BDNF levels using a human BDNF ELISA kit (Life Technologies Corporation Carlsbad, CA, USA). They were detected using a Biochrom Anthos Zenyth 200 rt Microplate Reader (Cambridge, UK).

### 2.7. Immunofluorescence Assay

The cells were fixed with 4% paraformaldehyde for 20 min, washed with phosphate-buffered saline (PBS), and incubated with Triton X-100 for 10 min (0.5% *v*:*v*). We performed blocking by incubating the cells with 5% (*w*:*v*) bovine serum albumin for 30 min. Following washing in PBS, the cells were incubated overnight with primary antibody at 4 °C. We evaluated the DNA damage using anti-8-hydroxy-2-deoxyguanosine (anti-8-OHdG, 1:1000; ab62623 Abcam, Cambridge, UK). Following their binding to primary antibodies, the cells were further incubated with Alexa Fluor-conjugated anti-rabbit, and anti-mouse (Jackson Immunoresearch, 1:1000) for 1 h. They were eventually stained and mounted using the Prolong^®^DiamondAntifade Mounting Medium, containing 4′,6-diamidino-2-phenylindole (Life Technology). We analyzed them with an Olympus DP71 device (100× and 200× magnification).

### 2.8. Mitochondrial Membrane Potential

THC-stimulated changes in the mitochondrial membrane potential (MMP) were assessed using the fluorescent reagent tetraethylbenzimidazolylcarbocyanine iodide (JC-1) with the JC-1-Mitochondrial Membrane Potential Assay Kit (Abcam, Cat. no. ab113850) following the manufacturer’s protocol. Cells were washed once with 1× dilution buffer and then incubated with 20 µM JC-1 dye in 1× dilution buffer for 10 min at 37 °C, protected from light. JC-1 dye was then removed, cells were washed once with 1× dilution buffer, and 100 µL of fresh 1× dilution buffer was added to each well. The red fluorescence in excitation (535 nm)/emission (590 nm) and green fluorescence excitation/emission (475 nm/530 nm) was measured using a Biochrom Anthos Zenyth 200 rt Microplate Reader (Cambridge, UK). Background fluorescence was subtracted from the fluorescence of treated cells, then the ratio of red (polarized) fluorescence divided by that of green (depolarized) fluorescence was obtained.

### 2.9. Co-Immunoprecipitation

The Catch and Release Reversible Immunoprecipitation System (Millipore) was used according to the manufacturer’s instructions. The proteins were eluted in 70 μL of the elution buffer and subjected to an immunoblotting analysis, using the anti-GSK-3β^S9^ and anti-p-Tau^S396^ antibodies.

### 2.10. Statistical Analyses

All measurements were produced at least three times under independent conditions. The results are shown as mean ± standard error of the mean (SEM). Statistics were analyzed with Mann-Whitney U-test. All statistical analyses were carried out on raw data using SPSS, version 20.0 (SPSS Inc, Chicago, IL, USA). * *p* value < 0.05 indicated a significant result. ** *p* < 0.01 indicated an extremely significant result.

## 3. Results

### 3.1. Mechanical Injury Induces Neuron Injury through Stretching, thus Altering the Mitochondrial Membrane Potential and Inducing Oxidative DNA Damage

**Figure 1:**
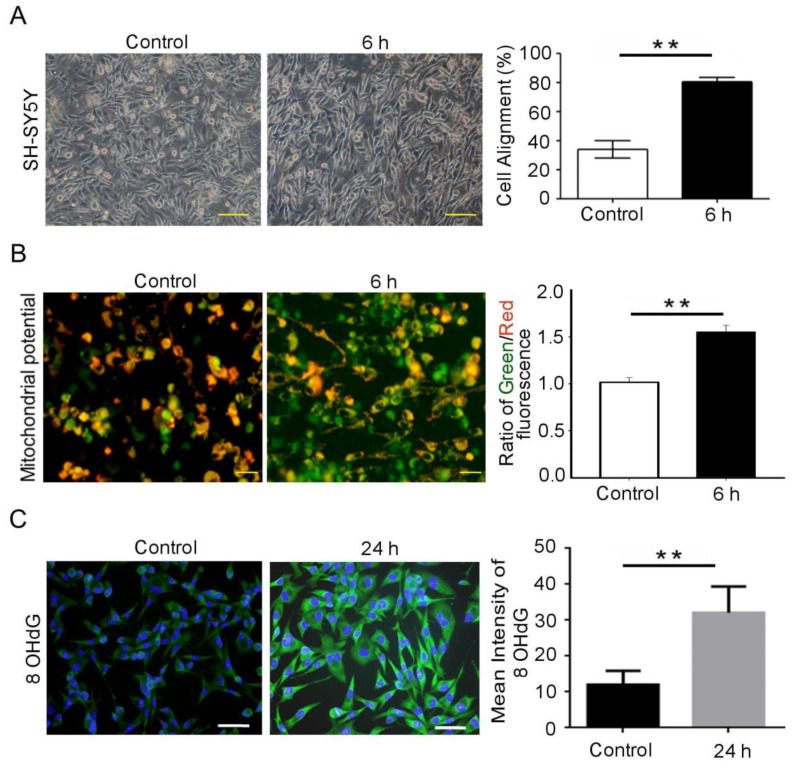

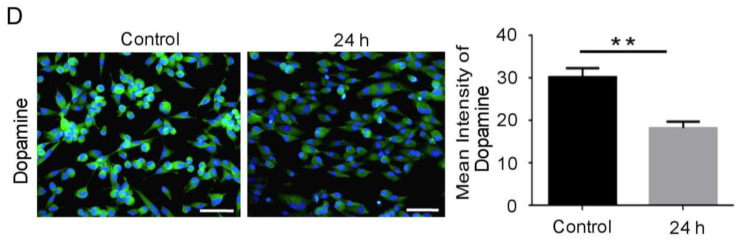
Mechanical injury induces neuron injury through stretching. (**A**) SH-SY5Y neuroblastoma cells are stretched in the uniaxial direction; cell alignment was measured by an angle deviation of 30° in plane, with both ends of each cell. Cell alignment after 6 h was measured using ImageJ. (**B**) The cells were stained with JC-1 probe immediately after 6 h of stretching. The JC-1 probe detects changes in the mitochondrial potential. It was analyzed by flow cytometry for 6 h after stretching. The mechanical stimulation altered the mitochondrial membrane potential. (**C**) A set of representative anti-8-OHdG immunofluorescent stained images (green) depicts an increase in 8-OHdG fluorescence signal at 24 h of stretching, compared to the control. (**D**) A set of representative anti-Dopamine immunofluorescent stained images (green) depicts a decrease in dopamine fluorescence signal at 24 h of stretching, compared to the control. Blue color 4′,6-diamidino-2-phenylindole denotes nuclei. Blue color 4′,6-diamidino-2-phenylindole denotes nuclei. Data are presented as mean ± SD. Scale bar = 50 μm; ** *p* < 0.01. 8-OHdG, anti-8-hydroxy-2-deoxyguanosine.

Neurons rely on signal transmission for cell-to-cell communication in a neuronal network. The mitochondrial membrane potential plays an important role in maintaining signal transduction in neurons. Herein, we investigated the effect of mechanical stress on neurons to establish an injured neuronal cell model. The neurons aligned perpendicularly to the stretching direction (Figure 1A). Furthermore, the MMP was altered after 6 h of cyclical stretching at 25% and 1 Hz uniaxial deformation, as shown by JC-1 staining. In turn, this alteration substantially increased the ratios of green/red fluorescence compared to those of the control. Thus, stretching triggers MMP collapse by depolarization (Figure 1B). TBI-induced oxidative stress in the brain can lead to neurodegenerative diseases as well. Oxidative stress is a major mediator of the secondary injury that follows TBI [29]. Neuronal death, a hallmark of TBI, where the loss of dopaminergic neurons and dopaminergic dysfunction is observed. Levels of dopamine (DA) were shown to be decreased 24 h after injury in an experimental mouse model of TBI [30]. Therefore, we determined to study if mechanical stress reduces levels of DA and oxidative DNA damage. Following stretching at 25% and 1 Hz for 24 h, the 8-OHdG immunoreactivity had substantially increased compared to the control (Figure 1C), and the levels of DA had substantially decreased compared to the control (Figure 1D). Hence, stretching-mediated mechanical injury altered the mitochondrial membrane potential and increased oxidative DNA damage.

### 3.2. BDNF Reduction Is Associated with Increased Amyloid-β/p-Tau

**Figure 2:**
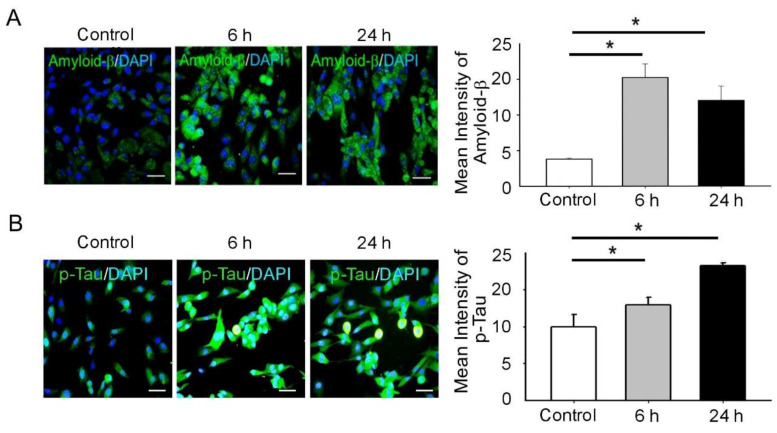

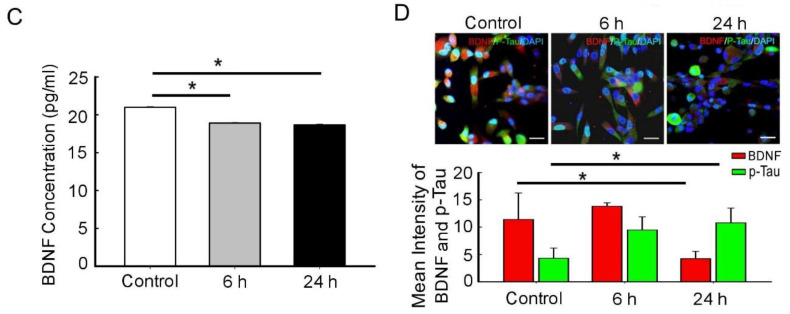
Mechanical injury through stretching upregulates amyloid β and p-Tau levels and decreases BDNF expression. (**A**,**B**) Representative green fluorescence images of amyloid β and p-Tau^S396^ positive cells in the SH-SY5Y, before and after stretching. The nuclei of the SH-SY5Y cells were counterstained with 4′,6-diamidino-2-phenylindole and exhibit blue fluorescence. Bar graphs representative of the amyloid β and p-Tau^S396^ fluorescence intensity in the SH-SY5Y cells of the indicated groups. The amyloid β and p-Tau^S396^ fluorescence intensity was significantly high in the 25%, 1 Hz group at 6 and 24 h. Scale bar = 200 μm. (**C**) An ELISA assay was performed to analyze BDNF expression. Mechanical injury significantly lowered BDNF levels, compared to the control. (**D**) Immunofluorescence assay was performed to analyze BDNF and p-Tau^S396^ protein expression. Bar graphs representative of the BDNF and p-Tau^S396^ fluorescence intensity in the SH-SY5Y cells of the indicated groups. The p-Tau^S396^ fluorescence intensity was significantly high in the 25%, 1 Hz group at 6 and 24 h. However, BDNF was attenuated at 24 h. The values are presented as means ± SEM (n = 6). Scale bar = 200 μm; * *p* < 0.05. BDNF, brain-derived neurotrophic factor; ELISA, enzyme-linked immunosorbent assay.

Mechanical stretching triggered neuronal injury by inducing oxidative DNA damage (Figure 1). The BDNF level, amyloid-β and p-Tau^s396,^ was altered following 24 h of cyclical stretching at 25% and 1 Hz uniaxial strain as shown by immunofluorescence and ELISA assay. Cyclical stretching decreased BDNF levels and increased amyloid-β/p-Taus396 in the SH-SY5Y neuroblastoma cells (Figure 2A–C). This BDNF decrease suggests that the neurons were being invoked by stress, whereby BDNF acted as a neuroprotective factor. Moreover, its decrease was accompanied by an increase in aggregated p-Tau^S396^ protein, which is an indication of neuronal injury (Figure 2D). Therefore, we could successfully establish a neuron injury model by mechanical stretching of the neuroblastoma cells.

### 3.3. Mechanical Injury through Stretching Upregulated p-GSK3β/p-Tau Protein Levels and Are Associated with a Reduction of the Insulin Pathway

**Figure 3:**
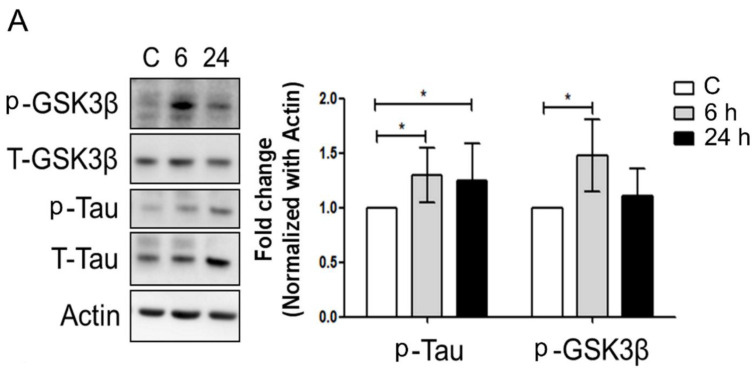

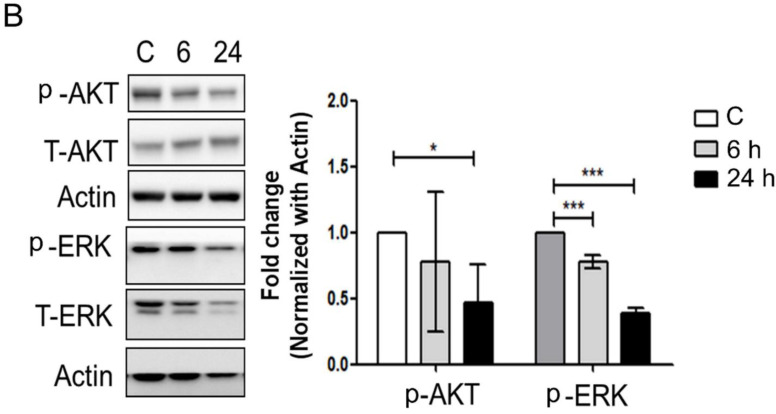
Mechanical injury through stretching upregulates p-Tau and p-GSK3β protein levels and decreases the insulin pathway. (**A**) p-Tau and p-GSK3β; (**B**) p-AKT and p-ERK; proteins expressions. p-GSK3β and p-Tau^S396^ protein levels significantly increased in the 25%, 1 Hz group at 6 and 24 h of stretching. However, the p-AKT and p-ERK protein expressions had the reverse effect. The values are presented means ± SEM (n = 6); *** *p* < 0.001; * *p* < 0.05. GSK3β, glycogen synthase kinase 3; ERK, extracellular-signal-regulated kinase.

Insulin/IGF resistance in AD brain results in decreased phosphoinositol-3-kinase (PI3K)/Akt [23,24] and increased activation of glycogen synthase kinase 3β (GSK-3β). Furthermore, injured neurons accumulate phosphorylated Tau and GSK3β. Thus, we aimed to determine if AKT/extracellular-signal-regulated kinase (ERK) signaling pathway defects upregulated amyloid-β/p-Tau^S396^. Both p-Tau^S396^ and p-GSK3β^S9^ protein levels were significantly upregulated, 6 h following the mechanical stretching (Figure 3A). p-AKT/p-ERK, the proteins were significantly downregulated following 6 and 24 h of mechanical stretching (Figure 3B). Thus, mechanical stretching downregulates the p-AKT/p-ERK and upregulates p-GSK3β/p-Tau protein levels, which results in neuronal injury.

### 3.4. The Interaction between GSK-3β and p-Tau Plays a Crucial Role and Is Associated with the Reduction of the Insulin Pathway

**Figure 4:**
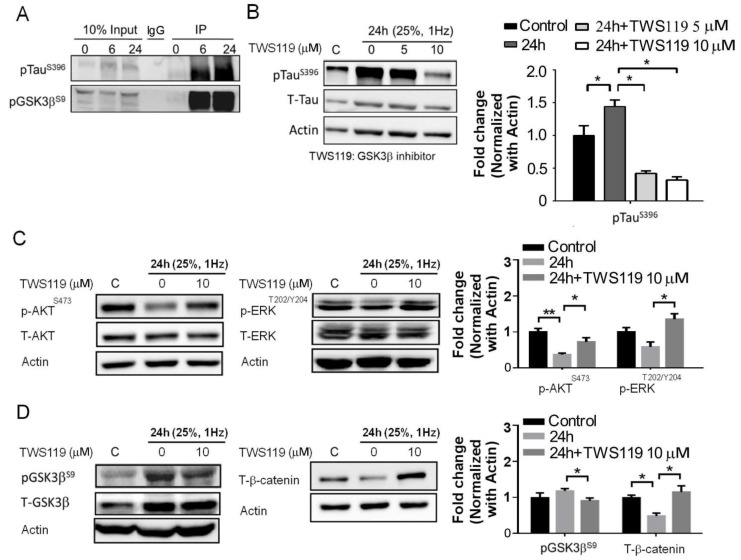
Mechanical injury promotes p-Tau-pGSK3β complex formation and affects the insulin pathway. (**A**) Co-immunoprecipitation was performed to study the p-Tau^S396^ and anti-GSK-3β^S9^ protein complex formation. The input protein comprises 10% of the total lysates, and the remaining 90% protein lysate was incubated with the p-Tau^S396^ or anti-GSK-3β^S9^ primary antibody. Co-immunoprecipitation was performed to study the p-Tau and p-GSK3β protein complex formation. The input protein comprises 10% of the total lysates, and the remaining 90% protein lysate was incubated with the p-GSK3β primary antibody. (**B**) We analyzed the function of p-GSK3β by adding the p-GSK3β inhibitor and TWS119 while the cells were being stretched. p-Tau^s396^ protein levels significantly decreased in the 25%, 1 Hz-TWS119 group. (**C**,**D**) We conducted Western blot to analyze phosphorylated p-Akt^S473^, p-ERK^T202/Y204^, p-GSK-3β^S9^, and T-β-catenin protein in SH-SY5Y cells, before and after the stretching or TWS119 administration. The immunoblot demonstrates greater levels of p-Akt^S473^, p-ERK^T202/Y204^, p-GSK-3β^S9^, and T-β-cateninin SH-SY5Y cells, before and after the stretching or TWS119 administration. ** *p* < 0.01; * *p* < 0.05. GSK3β, glycogen synthase kinase 3; ERK, extracellular-signal-regulated kinase.

**Figure 5:**
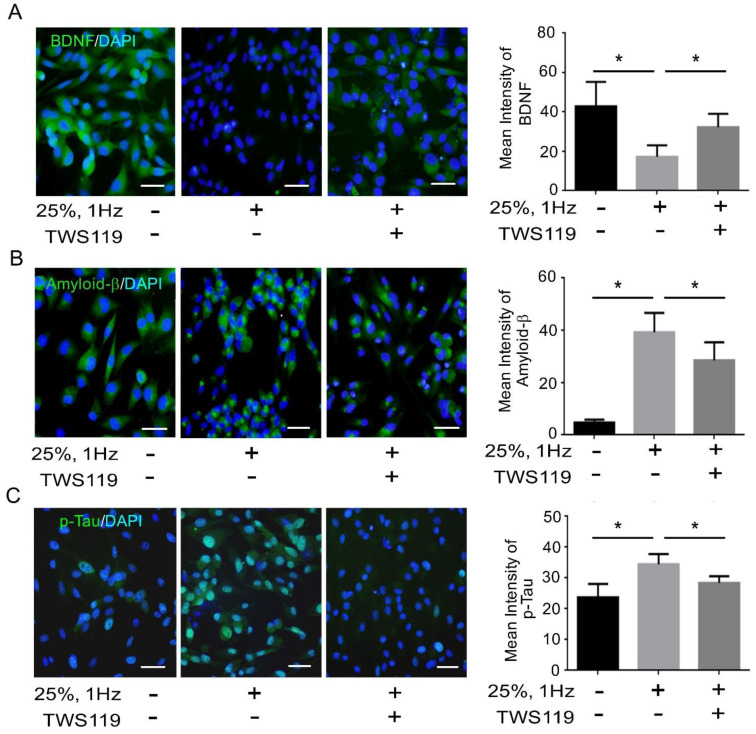
P-GSK3β reduces BDNF levels and upregulates amyloid β/p-Tau expression in the neuron injury model. (**A**–**C**) Representative green fluorescence images of BDNF, amyloid β, and p-Tau^S396^ positive cells in SH-SY5Y cells, before and after stretching or TWS119 administration. The nuclei of SH-SY5Y cells were counterstained with DAPI and exhibited blue fluorescence. Bar graphs are representative of the BDNF, amyloid β, and p-Tau^S396^ fluorescence intensity in the SH-SY5Y cells of the indicated groups. The BDNF fluorescence intensity increased substantially in the 25%, 1 Hz-TWS119 group, with diminished amyloid β and p-Tau^S396^ intensity. The values are presented as means ± SEM (*n* = 6); * *p* < 0.05. BDNF, brain-derived neurotrophic factor. Scale bar = 200 μm.

A defect in the insulin pathway is a critical link between Tau and/or Aβ pathologies that define AD [31]. The aforementioned upregulation of pGSK3β/p-Tau protein levels interfered with the insulin pathway. Therefore, we investigated the formation of p-Tau^S396^ and pGSK3β^S9^ protein complex by co-immunoprecipitation. The p-GSK3β^S9^ antibody was able to capture the p-Tau^S396^ protein following 6 and 24 h of mechanical stretching (Figure 4A). In addition, we determined the involvement of GSK-3β in the stretch-induced neuronal injury and insulin pathway. TWS119, the GSK3β phosphorylation inhibitor was administered to the cells at final concentrations of 0, 5, and 10 μM. In combination with 24 h stretching, the p-Tau^S396^ protein level was also downregulated when treated with 10 μM TWS119 (Figure 4B). The administration of TWS119 resulted in an increase in insulin signaling (Figure 4C). The p-GSK3β^S9^ protein level was downregulated when treated with 10 μM TWS119. However, β-catenin has the opposite effect (Figure 4D). Moreover, it leads to a decrease in Tau^S396^ phosphorylation and improves insulin receptor (IR) signaling [32]. Next, we examined the BDNF, Aβ, and Tau^S396^ phosphorylation levels. TWS119 administration resulted in an increase in BDNF levels and a decreased amyloid-β/p-Tau^s396^ in immunofluorescence (Figure 5A–C). Therefore, stretching-mediated mechanical injury downregulated the insulin pathway and upregulated p-GSK3β/p-Tau protein levels, which caused neuronal injury. However, TWS119 attenuates the mechanical stretching-induced activity of Aβ-Tau and improves neuronal injury. Hence, GSK3β promotes the expression of Aβ-Tau, which in turn downregulates IR signaling and BDNF production stretch-induced neuronal injury.

## 4. Discussion

TBI is defined as damage to the brain that consequently disrupts normal function. Previous in vitro studies on TBI used to stretch and shear forces on either rodent organotypic slices [33] or rudimentary single- or two-cell type cultures [34]. Despite being useful for studying axonal injury, the aforementioned models have limited translational relevance with regards to the extracellular matrix, stiffness, and cell–cell interactions that substantially influence the biophysical properties of mechanical injury in vivo [35]. The target signaling pathway for treating degenerative neuron disease is unclear. However, lost and damaged neurons cannot proliferate in our bodies. Hence, neurodegenerative diseases are usually progressive and are difficult to cure. The neurons generally do not move, are not stretched, or compressed. However, they experience a certain amount of strain during and even after TBI [9]. Considering their less motile behavior, a minor strain on the neurons can result in minor injury. The low motility behavior also implicates the significance of cell–cell communication between neurons. The smallest impact can also affect cell–cell communication by disrupting neuronal transmission.

Recently, researchers have attempted the development of in vitro TBI models using a bio-inspired mechanic device. Previous studies have used a high-frequency device to stretch the neurons [10]. Aggressive stretch can lead to pro-apoptotic neuron death and axonal damage. The neurons usually survive stretching-induced axonal damage, thus enabling a study of the injury to the axons [11]. Despite the role of stretching in promoting neuron growth, axonal growth did not necessarily result in axonal regeneration in previous studies. The axons repaired the damage; however, they were unable to regrow the entire length. Thus, stretch-induced neuronal injury can be used for studying neuron growth, death, and regeneration [12]. The diversity of neuropathology, combined with the heterogeneity in injury distribution, suggests an extensive pathological remodeling of the cellular microenvironment of the brain, the causes of which need to be elucidated to prevent TBI. Considering that mechanical forces are a primary contributor to the etiology of TBI, understanding the biomechanics of injury may elucidate complex injury mechanisms and explain the diverse pathophysiology associated with TBI. 

Neuronal death, a hallmark of TBI, is related to the development of neurodegenerative disorders such as Parkinson’s disease (PD), where the loss of dopaminergic neurons and dopaminergic dysfunction are observed. Hector et al., to simulate TBI subjected to 0%, 5%, 10%, 15%, 25% and 50% deformation, demonstrated that 24 h after injury, cell viability and apoptosis were determined by lactate dehydrogenase release and DNA fragmentation. Extracellular dopamine (DA) levels increased only at 50%. Levels of DA remained unchanged regardless of treatment. These data support the use of stretch as a model to simulate TBI in vitro in human dopaminergic neurons, replicating the acute effects of TBI in the dopaminergic system [13]. Besides, there is increasing evidence that apoptosis induced by TBI often starts with an accumulation of ROS and induces hypofunction of the striatal dopaminergic system in vitro and in vivo [34,36,37]. In this regard, SH-SY5Y cells retain dopamine-β-hydroxylase activity, thus being suitable for the neuromechanistic study [34,37]. Therefore, we determined to study if mechanical stress reduces levels of DA and oxidative DNA damage. The neurons aligned perpendicularly to the stretching direction (Figure 1A). Furthermore, the MMP was altered following 6 h of cyclical stretching at 25% and 1 Hz uniaxial strain as shown by JC-1 staining. This in turn substantially increased the ratios of green/red fluorescence than the control. Thus, stretching triggers MMP collapse by depolarization (Figure 1B). Following stretching at 25% and 1 Hz for 24 h, the 8-OHdG immunoreactivity had substantially increased, compared to the control (Figure 1C) and the levels of DA had substantially decreased, compared to the control (Figure 1D). Hence, stretching-mediated mechanical injury altered the mitochondrial membrane potential, DA release and increases oxidative DNA damage (Figure 1). Oxidative stress replicates the key transcriptional programs that are hallmarks of neuronal injury, including the upregulation of oxidative stress and aberrant phosphorylation of GSK-3β. Thus, the present founding determined that cyclical stretching increased in DNA damage downregulated BDNF release and increased amyloid-β/p-Tau^s396^ in the SH-SY5Y neuroblastoma cells (Figure 2A–C). This BDNF decrease suggests that the neurons were being invoked by a stress, whereby BDNF acts as a neuroprotective factor. Moreover, its decrease was accompanied with an increase in aggregated p-Tau^S396^ protein, which is an indication of neuronal injury (Figure 2D). In addition, the method accurately recapitulates two key pathological features of neurodegenerative diseases. The aggregation and accumulation of Aβ and Tau are the key pathological markers of TBI, which contribute to the progressive deterioration associated with TBI.

GSK3 is a key kinase contributing to abnormal phosphorylation of the microtubule-binding protein Tau in the process thought to cause neurofibrillary tangles in Alzheimer’s disease [38,39]. It is well known that the action of GSK3β is inhibited by phosphorylation of the enzyme on serine 9 following insulin activation [40]. However, insulin resistance causes inhibition of downstream signal transduction and GSK3 remains in its active form, which hyper-phosphorylates several substrates, including Tau protein, in nerve cells. Hyper-phosphorylation of Tau destabilizes the microtubules and induces neurofibrillary tangle formation that leads to AD [41]. Leng et al. demonstrated that GSK3β as a kinase required for high glucose-induced serine332 phosphorylation, ubiquitination, and degradation of IRS1 [42]. Besides, Liberman et al. studies identify Ser332 as the GSK-3 phosphorylation target in IRS-1, indicating its physiological relevance and demonstrating its attenuate insulin signaling [43]. Thus, GSK3β may play a causative role in the regulation of insulin pathway and connecting AD. Previous studies have established a correlation between AD an altered responsiveness to insulin and IGF stimulation in the brain [44]. Furthermore, the aforementioned abnormal accumulation of amyloid-β [45] and Tau aggregates may be linked to insulin/IGF resistance. Therefore, AD is presumably a metabolic disease [46]. Insulin/IGF signaling is responsible for regulating neuronal growth, survival, differentiation, migration, energy metabolism, cytoskeletal assembly, synapse formation, neurotransmitter function, and plasticity [47]. Insulin/IGF resistance in the brain can result in the inhibition of pro-survival and pro-growth signaling pathways. Furthermore, insulin and IGF bind to their own receptors on the cell membrane, leading to the phosphorylation of insulin receptor substrate (IRS) molecules, which eventually activate downstream signals. Accumulating evidence indicates that BDNF and its interaction with ROS may be crucial for neurodegenerative and neuropsychiatric conditions [48]. However, there is no therapeutic approach that appears promising in reducing the progression of TBI to chronic neurodegenerative disease. Increased phosphorylation of Tau was previously correlated to neurodegeneration, in the context of TBI in a rodent model [49] and in numerous clinical studies [5]. Furthermore, we also founding that injured neurons through mechanical stretching accumulate phosphorylated Tau and GSK3β. Both p-TauS396 and p-GSK3βS9 protein levels were significantly upregulated, 6 h following the mechanical stretching (Figure 3A). Besides, p-AKT/p-ERK proteins of survival and cell growth were significantly downregulated following 6 and 24 h of mechanical stretching (Figure 3B). Thus, the present result also demonstrated that stretching-mediated mechanical injury downregulated the insulin pathway and upregulated p-GSK3β/p-Tau protein levels, which caused neuronal injury.

Multiple lines of evidence strongly suggest that the inhibition of GSK-3 is a potential target for the treatment of TBI. GSK-3 constitutively inhibits neuroprotective processes and promotes apoptosis. Following TBI, the receptor tyrosine kinase (RTK) and canonical Wnt signaling pathways inhibit GSK-3 as an innate neuroprotective mechanism against TBI. GSK-3 inhibition via GSK-3 inhibitors and RTK-activating drugs or Wnt signaling is likely to reinforce the innate neuroprotective mechanism. GSK-3 inhibition studies using rodent TBI models have demonstrated that this inhibition produces diverse neuroprotective actions, such as a reduction in the size of the traumatic injury, Tauopathy, Aβ accumulation, and neuronal death, caused by the release and activation of neuroprotective substrates. The above-mentioned effects are correlated with reduced TBI-induced behavioral and cognitive symptoms [50]. We investigated the formation of p-Tau^S396^ and pGSK3β^S9^ protein complex by co-immunoprecipitation. The p-GSK3β^S9^ antibody was able to capture the p-Tau^S396^ protein following 6 and 24 h of mechanical stretching (Figure 4A). In addition, we determined the involvement of GSK-3β in the stretch-induced neuronal injury and insulin pathway. TWS119, the GSK3β phosphorylation inhibitor was administered to the cells at final concentrations of 0, 5, and 10 μM, in combination with 24 h stretching, the p-Tau^S396^ protein level was also downregulated when treated with 10 μM TWS119 (Figure 4B). The administration of TWS119 resulted in an increase in the insulin signaling (Figure 4C). Moreover, it leads to a decrease in Tau^S396^ phosphorylation and improved insulin receptor (IR) signaling. However, TWS119 attenuates the mechanical stretching-induced activity of Aβ-Tau and improves neuronal injury. Hence, GSK3β promotes the expression of Aβ-Tau, which in turn downregulates IR signaling and BDNF production in stretch-induced neuronal injury (Figure 5).

**Figure 6:**
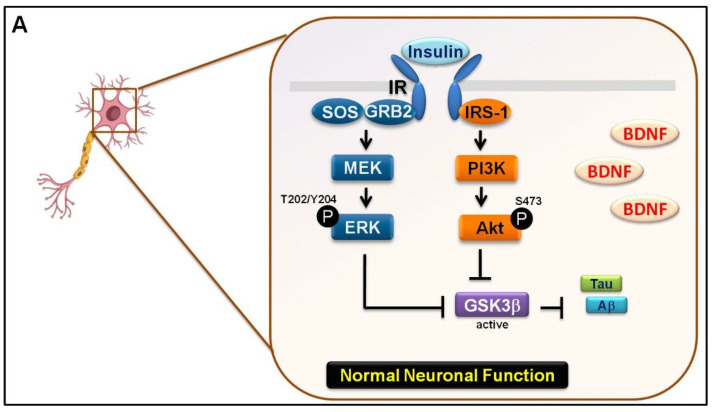

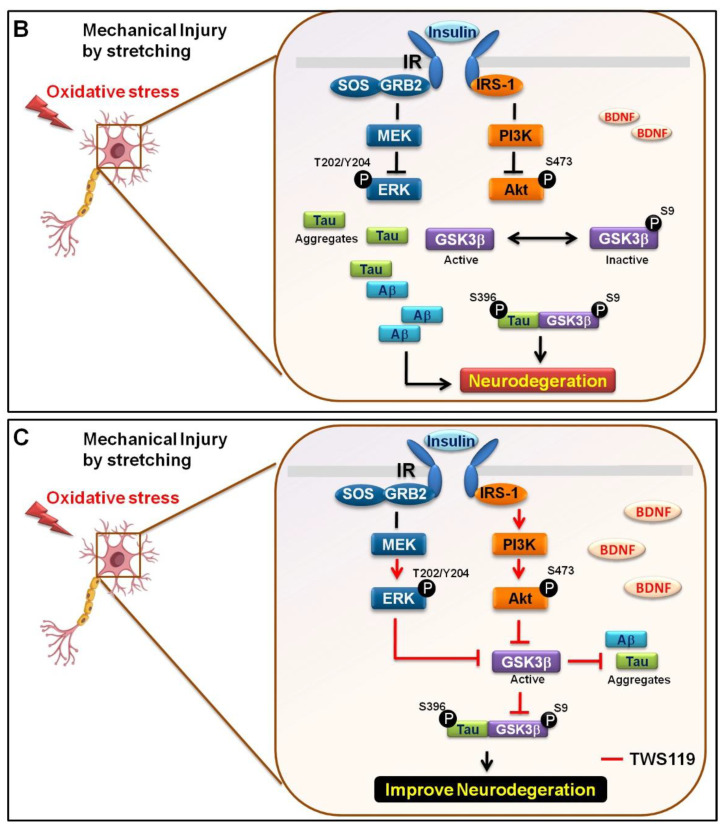
A schematic model suggesting the mechanistic details of the regulation of neurodegenerative diseases by GSK-3β. (**A**) Under normal conditions, the activated PI3K/Akt ERKs signaling pathway leads to the inactivation of GSK-3β, which suppresses the downstream phosphorylation of Tau and Aβ that results in positive regulation of cognitive signaling, such as BDNF and insulin. (**B**) Under pathological conditions, mechanical stretching increases GSK-3β activity, induces oxidative stress, and increases MMP. Upregulation of GSK-3β activity negatively regulates BDNF and insulin signaling and promotes phosphorylation events critical to the development of Aβ and Tau aggregation. (**C**) Tws119, an inhibitor of GSK-3β, improves pathological conditions by positively regulating BDNF and insulin signaling. Moreover, it reduces phosphorylation events critical to the development of Aβ and Tau aggregation. BDNF, brain-derived neurotrophic factor; MMP, mitochondrial membrane potential; and GSK-3, glycogen synthase kinase 3.

Mechanical stretch injury, which was developed and characterized by Ellis and coworkers, had been used to study the effects of trauma on neurons and astrocytes in vitro [51,52]. Following TBI, neuronal loss is characterized by oxidative stress reaction, mitochondrial dysfunction, neurotoxicity, and neuroinflammation [53,54]. Our findings demonstrate that mechanical stretch injury to SH-SY5Y cells resulted in oxidative stress and the production of MMP that triggered insulin signaling defects and events critical for Aβ and Tau aggregation mimicking pathological conditions. In addition, the neuronal insulin signaling defects promoted the formation of pTau^S396^ and pGSK-3β^S9^ complex, thus further implying the neuronal stress. Thus, inhibition of GSK-3β activity can serve as a potential target for increasing BDNF levels and insulin signaling as well as reducing phosphorylation events, which are critical for Aβ and Tau aggregation. Tws119, an inhibitor of GSK-3β, improves pathological conditions, by positively regulating BDNF and insulin signaling. Overall, the aforementioned neuronal insulin signaling defect is a core mechanism that induces the formation of pTau^S396^ and pGSK-3β^S9^ complex. Hence, the neurons under stress may alleviate this defect upon the inactivation of the GSK-3β (Figure 6). The present findings suggest that the protective role of GSK3β in SH-SY5Y cells that were exposed to mechanical stretch injury was mainly due to its cause insulin signaling defect and Aβ and Tau aggregation. Therefore, our study has some limitations such as SH-SY5Y is a proliferative cell line, which is entirely different from the terminally differentiated neurons. Although SH-SY5Y cells exhibit some features of neurons [37], primary cultured neurons might be more appropriate for investigating endogenous mechanisms after injury.

## 5. Conclusions

This is the first study to demonstrate the mechanism by which mechanical stretching stimulates the accumulation of amyloid-β, a neuronal injury biomarker through p-Tau^S396^ and pGSK3β^S9^ protein complex formation. The latter, in turn, downregulates IR signaling and BDNF production. The role of mechanical stretching on neurons requires further investigation to explore the upstream mechanism underlying amyloid-β accumulation. This neuron injury model was developed to mimic neurons under stress. Mechanical stretching does not normally occur on neurons in vivo. Nonetheless, they respond to mechanical cues for intracellular signaling. Therefore, the neuron injury cell model can explain the mechanistic signaling for potential drug discovery in targeting the accumulated amyloid-β, p-Tau^S396^, and pGSK3β^S9^ protein complex formation.

## Data Availability

All data generated or analysed during this study are included in this published article.
